# Marfan-Like Clinical Features in DLG4 (Discs Large MAGUK Scaffold Protein 4)-Related Synaptopathy: A Brazilian Case Report

**DOI:** 10.7759/cureus.114477

**Published:** 2026-08-13

**Authors:** Julia Giraudon, Anna Carolina De Rizzo Cantoni Rosa, Maria Fernanda Vieira Fernandes, Nilton Salles Rosa Neto

**Affiliations:** 1 Rheumatology, Universidade Santo Amaro, São Paulo, BRA; 2 Medicine, Universidade Santo Amaro, São Paulo, BRA; 3 Center for Rare Diseases and Immunology, Hospital Nove de Julho, São Paulo, BRA

**Keywords:** case report, dlg4, marfanoid features, psd-95, synaptopathy

## Abstract

*DLG4*-related synaptopathy is a rare neurodevelopmental condition caused by pathogenic variants in the *DLG4 *gene, which encodes the postsynaptic density protein 95 (PSD-95). The disorder is characterized by a broad clinical spectrum that may include intellectual disability, autism spectrum disorder (ASD), epilepsy, hypotonia, sleep disturbances, and marfanoid manifestations. Most reported cases arise from de novo variants, although autosomal dominant inheritance with variable phenotypic expressivity has also been described. This report describes a 21-year-old man presenting with chronic musculoskeletal pain, sensory hypersensitivity, tremors, cognitive decline, ASD traits, psychotic manifestations, and marfanoid features. Additional symptoms included gastroesophageal reflux, dysphagia, constipation, urinary hesitancy with interrupted urinary stream, nocturnal polyuria, visual complaints, and impaired sleep quality. Cytogenetic analysis demonstrated a normal male karyotype (46,XY). Whole-exome sequencing identified a heterozygous missense variant of uncertain significance (VUS) (c.149A>T; p.Tyr50Phe) in the *DLG4* gene. Although the patient’s phenotype shows significant overlap with previously described presentations of *DLG4*-related synaptopathy, the variant is currently classified as a VUS and its clinical significance remains undetermined. He continues to undergo multidisciplinary follow-up and remains on pregabalin therapy for symptomatic pain control. This report further broadens the recognized phenotypic spectrum potentially associated with *DLG4*-related synaptopathy, highlighting the disorder's clinical heterogeneity while acknowledging that the identified variant remains of uncertain significance and therefore limits definitive genotype-phenotype correlation. Clinicians should consider this diagnosis in individuals presenting with neurodevelopmental manifestations, sensory disturbances, and marfanoid features. Early recognition and comprehensive genetic evaluation may support diagnostic clarification, guide management, and improve clinical outcomes.

## Introduction

Discs large MAGUK scaffold protein 4 (DLG4)-related synaptopathy belongs to a heterogeneous group of neurodevelopmental disorders known as synaptopathies, a rare condition with no well-established population prevalence. These disorders are characterized by structural and functional alterations in synapses that impair neuronal plasticity and synaptic transmission [1]. Synaptopathies result from disruption of the molecular machinery that governs synaptic development, organization, and plasticity. Normal synaptic function depends on the coordinated activity of pre- and postsynaptic proteins, as well as interactions with glial cells and the extracellular matrix, which provide structural support and regulate synaptic signaling. As many synaptic proteins are also expressed in non-neuronal tissues, disturbances may extend beyond the nervous system, contributing to connective tissue abnormalities and skeletal manifestations [2].

Among the genes involved, DLG4 encodes the postsynaptic density protein 95 (PSD-95), a key component of the postsynaptic density of glutamatergic neurons [1]. This protein plays a central role in anchoring and modulating N-methyl-D-aspartate (NMDA) and α-amino-3-hydroxy-5-methyl-4-isoxazolepropionic acid (AMPA) receptors and is essential for synaptic homeostasis, memory consolidation, and higher cognitive processes [1]. Pathogenic variants in DLG4 are associated with a spectrum of neurodevelopmental disorders characterized by intellectual disability, autism spectrum disorder (ASD), epilepsy, sleep disturbances, hypotonia, ataxia, and neurodevelopmental regression [3]. These variants lead to PSD-95 dysfunction, impairing synaptic organization and glutamatergic signaling [4]. In addition, PSD-95 dysfunction may affect interactions with Shaker-type potassium channels (Kv1), ionotropic glutamate receptors, and cell adhesion proteins, further contributing to the clinical phenotype [4].

DLG4-related synaptopathy is rare, underdiagnosed, and still poorly characterized, with fewer than 100 cases reported [5]. Variants occur predominantly de novo, although autosomal dominant inheritance has also been described [6]. The phenotype typically manifests early in childhood with global developmental delay, cognitive regression, and often refractory epilepsy, and may be accompanied by behavioral and neuropsychiatric dysfunction [7]. In addition to central nervous system involvement, oculomotor abnormalities such as strabismus and nystagmus, as well as skeletal alterations including marfanoid features, have been reported [1].

Marfanoid habitus refers to individuals who exhibit skeletal features suggestive of Marfan syndrome but do not meet the established diagnostic criteria for the disorder. Clinical presentations are highly heterogeneous, and unlike classic Marfan syndrome, many affected individuals also present with intellectual disability, a phenotype referred to as marfanoid habitus with intellectual disability (MHID). MHID is genetically complex, with many cases remaining molecularly unexplained. In addition to the well-recognized syndromes caused by variants in *MED12*, *ZDHHC9 *and *UPF3B *(Lujan-Fryns syndrome) and *SKI *(Shprintzen-Goldberg syndrome), pathogenic variants in *DLG4, NFIX, EHMT1, ZEB2*, and *ATP1A1 *have also been implicated in individuals with MHID, further expanding its genetic heterogeneity. Understanding the shared molecular pathways underlying these disorders may help explain their overlapping and distinct clinical features, ultimately improving diagnosis, genotype-phenotype correlations, and patient management [8].

Given the insidious nature of DLG4-related synaptopathy and the nonspecific presentation of its early manifestations, underrecognition remains a challenge. The description of additional cases is essential to expand the phenotypic spectrum and identify clinical and genetic markers that may improve diagnostic accuracy. In this context, including DLG4 in the genetic evaluation of individuals with hypermobility spectrum disorders or marfanoid habitus, particularly when associated with neurodevelopmental or neuropsychiatric features, may facilitate identification of additional cases. The present report describes a Brazilian patient with a phenotype highly consistent with DLG4-related synaptopathy, expanding the phenotypic spectrum potentially associated with this condition and underscoring its clinical heterogeneity.

## Case presentation

The present case report was prepared in accordance with the 2013 CAse REports (CARE) checklist. It was approved by the Research Ethics Committee of Universidade Santo Amaro, São Paulo, Brazil (approval number 7,433,349; CAAE 85704124.4.0000.0081). A 21-year-old man was evaluated for a complex multisystem disorder involving neurodevelopmental, neuropsychiatric, sensory, musculoskeletal, gastrointestinal, urinary, and connective tissue disorder-like manifestations. He was born prematurely at approximately seven months of gestation by cesarean section after a high-risk pregnancy complicated by maternal preeclampsia and postpartum hemorrhage. Independent walking and the production of first spoken words occurred at approximately two years of age. Although he remained academically functional and never repeated a school year, he later perceived a gradual decline in cognitive performance, particularly in written communication, word retrieval, learning, and processing speed.

Since childhood, he had experienced difficulties with social communication and reciprocity, behavioral rigidity, limited adaptability to changes, marked intolerance to noise, sensory hypersensitivity, discomfort with physical contact initiated by others, and restricted emotional expression. These characteristics were considered consistent with autism spectrum disorder traits. His psychosocial history was also notable for bullying, emotional distress, and social isolation. Family history included maternal cerebral arteriovenous malformation, osteopenia, and hormonal abnormalities. No parental consanguinity was reported, and familial segregation testing for the identified DLG4 variant was not available.

At 16 years of age, he developed chronic diffuse musculoskeletal pain that initially affected the hands and subsequently spread throughout the body. The pain was exacerbated by emotional stress and temperature fluctuations, resulting in difficulty writing and performing routine activities. Additional complaints included joint pain without perceived swelling or inflammatory skin changes, persistent fatigue despite rest, tremors during periods of more intense pain, muscle stiffness, a subjective sensation of slowed movement, and recurrent loss of balance. Alongside these manifestations, he described marked hypersensitivity to touch, heat, clothing friction, noise, and other external stimuli.

The neuropsychiatric history included longstanding depressed mood, anhedonia, suicidal ideation, behavioral dysregulation, and occasional visual and tactile hallucination-like experiences. Cognitive complaints consisted of slowed thought processing, confusion between words, impaired written expression, and reduced learning performance, all of which became more pronounced during periods of pain and fatigue. Sleep disturbances had also been present since childhood and were characterized by light, nonrestorative sleep, recurrent awakenings, and episodes of sleep paralysis, with nocturnal awakenings frequently associated with pain.

Approximately one year before the most recent evaluation, he developed episodic pulsatile headaches lasting several hours, accompanied by photophobia, right-sided ocular burning, nausea, and fluctuating blurred vision at both near and far distances. Bright light acted as both a trigger and an aggravating factor. Despite these symptoms, he had never used corrective lenses or received treatment for visual impairment.

Recurrent skin lesions were also reported after sun exposure, predominantly affecting the trunk and upper limbs. They were described as hypopigmented, scaly, occasionally blistering, and mildly pruritic, with associated burning and increased tactile sensitivity. These symptoms were aggravated by heat and friction.

Gastrointestinal complaints included recurrent epigastric pain described as stabbing, sharp, or burning. The pain was exacerbated by meals and deep inspiration and occasionally radiated to the lower back. Associated symptoms included gastroesophageal reflux (GERD), frequent heartburn, early satiety, chronic constipation occasionally accompanied by mucus, and mild intermittent dysphagia. Choking episodes, including with liquids, led him to voluntarily reduce his food intake. In addition, motion frequently triggered intense nausea, sometimes followed by vomiting, blurred vision, and a rotational sensation of dizziness. Persistent taste disturbances and hypogeusia were also reported. Urinary manifestations included polyuria, nocturnal urinary frequency, occasional dysuria, hesitancy, and an intermittently interrupted urinary stream. According to the clinical history, the gastrointestinal and urinary complaints became more prominent as the neurological, sensory, and pain-related manifestations worsened.

**Figure 1:**
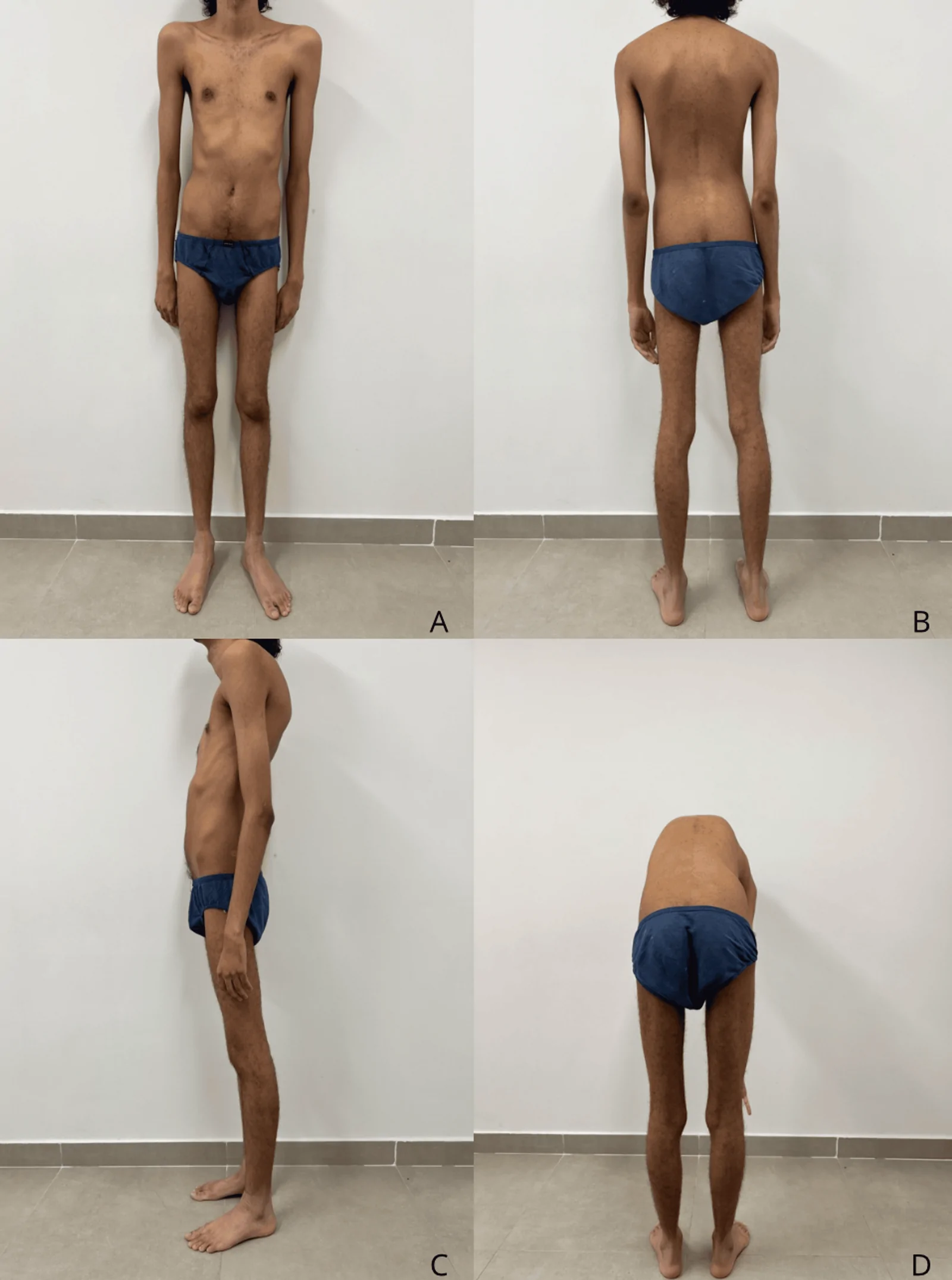
General phenotypic aspects of the patient. A: Anterior view in orthostasis, showing a long, lean biotype and excavated thorax. B: Posterior view with asymmetrical shoulders and slender limbs. C: Lateral view demonstrating thoracic hyperkyphosis and anterior trunk projection. D: Trunk flexion revealing asymmetrical arching compatible with mild scoliosis.

**Figure 2:**
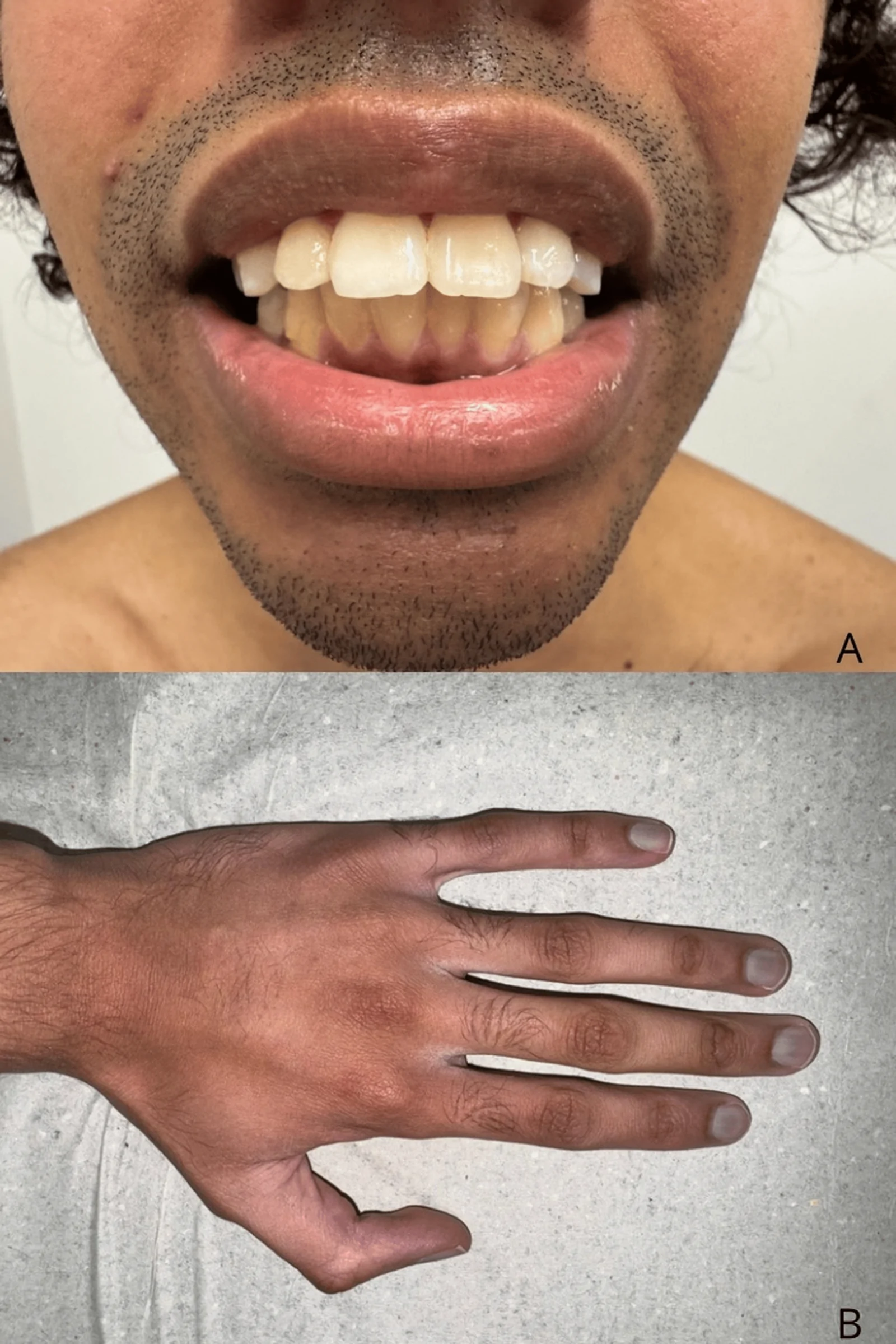
Additional physical findings. A: Frontal view of the oral cavity showing lower dental overlap and malocclusion. B: Right hand with long, tapered fingers and nails with a diffuse purplish color.

Physical examination revealed a marfanoid habitus characterized by dolichostenomelia, pectus excavatum, thoracic hyperkyphosis, and mild anterior projection of the trunk. On forward flexion, an asymmetric spinal curvature suggestive of mild-to-moderate scoliosis was observed. Examination of the hands showed long, thin, tapering fingers consistent with arachnodactyly. Diffuse violaceous discoloration of the nails was also noted, although nail shape and thickness were preserved. Oral examination demonstrated lower dental crowding, irregular alignment of the dental arch, and malocclusion. The clinical photographs illustrating the skeletal and postural findings are presented in Figures 1, 2.

As part of the diagnostic investigation, a skin biopsy obtained from the abdominal region on October 24, 2019, demonstrated chronic superficial perivascular dermatitis, with a mild inflammatory infiltrate in the superficial dermis, scattered melanophages, and mild melanin deposition. These findings were interpreted as residual post-inflammatory hyperpigmentation. Computed tomography of the abdomen was subsequently performed on March 13, 2020, because of upper abdominal pain, weight loss, and episodes of hematuria. The examination demonstrated narrowing of the left renal vein at the aortomesenteric portion with proximal dilatation, consistent with Nutcracker syndrome.

Conventional G-banded karyotyping at a resolution of 500 bands demonstrated a normal male karyotype (46,XY), without detectable numerical or structural chromosomal abnormalities. Whole-exome sequencing (WES) subsequently identified a heterozygous missense variant of uncertain significance in DLG4, c.149A>T (p.Tyr50Phe). WES was performed at the Eurofins Centro de Genomas (Genoma Center), São Paulo, Brazil, following the laboratory's standard protocols for library preparation, sequencing, and bioinformatic analysis. According to the Genome Aggregation Database version 4.1.1, the variant had an allele count of 27 among 1,607,076 analyzed alleles, corresponding to an allele frequency of 0.00001680 (1.68 × 10^-^⁵).

Computational predictions regarding the potential effect of the variant were conflicting. The Combined Annotation Dependent Depletion score was 23.8, and the affected nucleotide showed high evolutionary conservation, with a phyloP score of 6.33. In contrast, the Rare Exome Variant Ensemble Learner score was 0.214, and PolyPhen-2 classified the substitution as benign, with a score of 0.010. Similarly, SpliceAI and Pangolin did not predict a clinically significant effect on RNA splicing, with scores of 0.020 and -0.070, respectively. The variant was absent from ClinVar at the time of evaluation. Collectively, these findings were considered insufficient to establish pathogenicity or provide definitive molecular confirmation [9].

Features considered to overlap with the previously reported DLG4-related phenotype included developmental delay, ASD-related traits, cognitive impairment, sleep disturbances, sensory abnormalities, and marfanoid skeletal findings. In contrast, chronic diffuse pain, gastrointestinal and urinary complaints, dermatologic abnormalities, nail discoloration, and Nutcracker syndrome are less consistently described in association with DLG4 and should therefore be considered potentially phenotype-expanding observations rather than established manifestations of the disorder.

Regarding treatment, he underwent physiotherapy twice weekly for approximately one year, with partial improvement in musculoskeletal symptoms and functional limitations. Common analgesics, including ketorolac-based medications, provided limited relief. At the most recent evaluation, he was receiving pregabalin at a total daily dose of 225 mg for pain and sensory symptoms, as well as vitamin D supplementation at 7,000 IU weekly. Risperidone at 1 mg/day had been introduced approximately two months earlier because of behavioral dysregulation, hallucination-like experiences, sensory disturbances, and sleep impairment.

Pregabalin and risperidone were generally well tolerated, without major reported adverse effects. Treatment resulted in partial improvement in pain, sensory symptoms, emotional stability, behavioral manifestations, and sleep quality. Nevertheless, substantial functional impairment and multisystem symptoms persisted.

The progressive and multisystem nature of the presentation resulted in evaluations across several medical specialties over multiple years. Diagnostic clarification was complicated by the rarity and broad phenotypic spectrum of DLG4-related synaptopathy, its overlap with connective tissue disorder-like and neuropsychiatric phenotypes, and the classification of the identified DLG4 variant as a variant of uncertain significance.

Chronic pain, sensory hypersensitivity, and cognitive difficulties were described as the principal factors affecting his daily functioning, emotional well-being, and social participation. He reported that multidisciplinary follow-up and the possibility of a unifying diagnostic explanation reduced some of the uncertainty associated with his prolonged diagnostic journey. Family support and symptom-oriented treatment were considered important for maintaining daily functioning and emotional stability.

He remains under rheumatologic and multidisciplinary follow-up for monitoring of his musculoskeletal, neurodevelopmental, neuropsychiatric, sensory, gastrointestinal, and urinary manifestations. Based on the overall phenotypic overlap, DLG4-related synaptopathy remains the leading diagnostic hypothesis. However, the DLG4 variant cannot currently be considered causative, and the diagnosis has not been molecularly confirmed.

**Figure 3:**
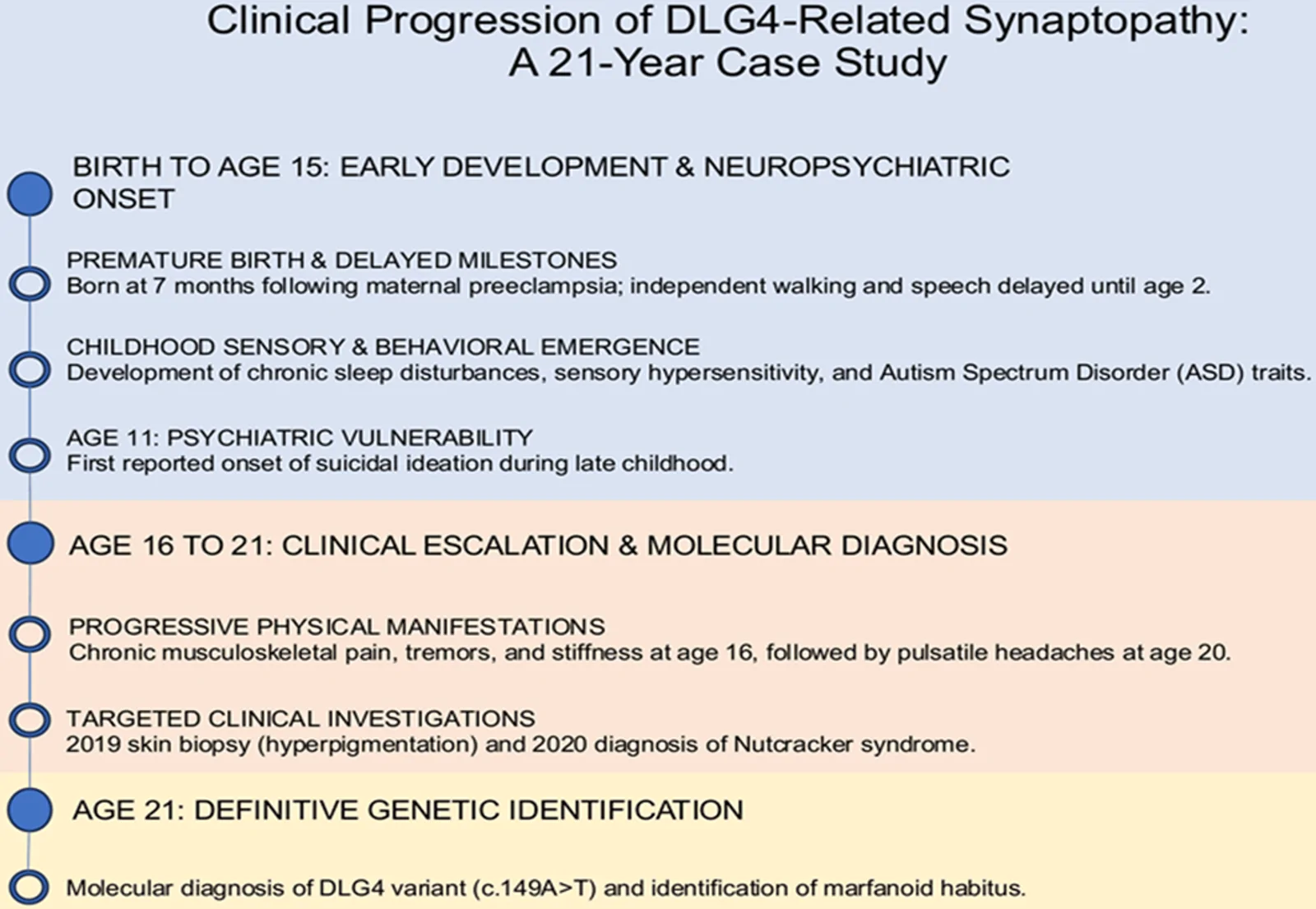
Timeline of clinical manifestations, diagnostic evaluation, and therapeutic interventions. Chronological summary of the patient’s neurodevelopmental history, progression of systemic manifestations, diagnostic investigations, and multidisciplinary management. Created in Microsoft PowerPoint, version 2.112.3 (Microsoft Corporation, Redmond, WA, USA). DLG4: discs large MAGUK scaffold protein 4.

At the most recent assessment, he was clinically stable, without evidence of substantial progression, although persistent symptoms continued to require specialized longitudinal care. Because no disease-specific therapy is currently available, management remains symptomatic and supportive, with emphasis on pain control, behavioral and sensory symptoms, sleep quality, preservation of functional capacity, and coordinated multidisciplinary follow-up. A chronological summary of the patient’s clinical course, diagnostic investigations, and therapeutic interventions is presented in Figure 3.

## Discussion

We report the case of a young adult with a complex phenotype beginning in childhood, characterized by chronic musculoskeletal pain, sensory abnormalities, progressive cognitive impairment, behavioral and neuropsychiatric manifestations, sleep disturbances, and marfanoid features. Because chronic musculoskeletal pain and functional limitation were the predominant presenting complaints, the patient was referred for rheumatologic evaluation. The unusual combination of connective tissue disorder-like features, neurodevelopmental impairment, neuropsychiatric manifestations, and multisystem involvement raised suspicion of an underlying monogenic disorder. Whole-exome sequencing subsequently identified a heterozygous missense variant of uncertain significance in the *DLG4* gene. Although the variant remains insufficient to establish molecular causality, the patient's phenotype shows remarkable overlap with previously described cases of *DLG4*-related synaptopathy. Table 1 illustrates this comparison, summarizing the clinical features of the reported patient in contrast with the frequencies of manifestations documented in literature-based of DLG4-related synaptopathy.

**Table 1:** Comparison of clinical features between the reported patient and literature-based of DLG4-related synaptopathy. Summary of the phenotypic spectrum and frequency of clinical manifestations associated with DLG4-related synaptopathy. Literature frequencies represent consolidated data from published cohorts, specifically drawing from references [2,3,5]. ASD, Autism Spectrum Disorder; GERD, Gastroesophageal Reflux Disease.

Clinical Feature	Frequency in Literature	Findings in Reported Patient (Present Study)
Intellectual Disability	~98%-100%	Yes (Progressive cognitive difficulties and decline)
Developmental Delay	84%	Yes (Milestones achieved at ~2 years of age)
Autism Spectrum Disorder	56%	Yes (ASD traits and social communication difficulties)
Epilepsy / Seizures	53%	No (To date)
Sleep Disturbances	45%	Yes (Light sleep, nocturnal awakenings, and sleep paralysis)
Marfanoid Habitus	24%	Yes (Prominent: pectus excavatum, arachnodactyly, and scoliosis)
Movement Disorders	46%	Yes (Tremors, muscle stiffness, and loss of balance)
Cognitive Regression	33%-40%	Yes (Late-onset regression: word confusion and slowed thinking)
Gastrointestinal Issues	29%	Yes (GERD, dysphagia, and chronic constipation)
Vision/Ocular Findings	50%	Yes (Blurred vision and photophobia)

This case therefore emphasizes the potential role of rheumatologic assessment in the investigation of patients with unexplained chronic pain and connective tissue manifestations, particularly when these findings coexist with cognitive, behavioral, sensory, or psychiatric abnormalities, and highlights the importance of a comprehensive differential diagnosis in individuals presenting with marfanoid phenotypes and overlapping neuropsychiatric features. *DLG4*-related synaptopathy has been associated with heterozygous variants that alter the expression or function of PSD-95, a postsynaptic scaffolding protein with a central role in cytoskeletal organization, glutamatergic signaling, synaptic plasticity, and neuronal maturation [1]. The clinical spectrum reported in the literature is broad and includes developmental delay, intellectual disability, autism spectrum disorder, epilepsy, sleep disturbances, hypotonia, oculomotor abnormalities, and marfanoid features [3,4], several of which were also observed in the present case. In particular, the patient’s ASD-related traits, progressive cognitive difficulties, hallucination-like experiences, sensory abnormalities, visual complaints, gastrointestinal manifestations, scoliosis, and pectus excavatum show considerable overlap with previously reported phenotypes [4,6], supporting the clinical hypothesis of *DLG4*-related synaptopathy despite the current classification of the identified molecular finding as a variant of uncertain significance.

Although most pathogenic *DLG4* variants reported to date have arisen de novo, cases demonstrating autosomal dominant inheritance with variable expressivity have increasingly been recognized, and recent studies indicate that first-degree relatives may exhibit milder, isolated, or atypical manifestations [10,11]. This broad and heterogeneous phenotypic spectrum may contribute to fragmented care across multiple specialties, including neurology, psychiatry, medical genetics, and potentially rheumatology, particularly when musculoskeletal and connective tissue disorder-like findings predominate. Recognition of recurring clinical patterns, such as musculoskeletal pain associated with functional limitation, sensory disturbances, ASD-related features, cognitive impairment, and marfanoid characteristics, may therefore promote earlier diagnostic suspicion, facilitate coordination among specialists, and guide appropriate molecular and familial evaluation [1,3].

The differential diagnosis of *DLG4*-related synaptopathy is extensive because its manifestations overlap with those of several nonspecific syndromic intellectual developmental disorders, making molecular testing essential for diagnostic clarification [3]. Other synaptopathies should be considered, particularly SYNGAP1-related intellectual disability, as well as disorders associated with intellectual, language, or developmental regression, including Rett syndrome and Landau-Kleffner syndrome [5]. When a marfanoid habitus is present, the investigation should also encompass connective tissue and marfanoid syndromes such as Marfan syndrome, Loeys-Dietz syndrome, Shprintzen-Goldberg syndrome, and Snyder-Robinson syndrome [4,6]. More broadly, the diagnostic approach should include autosomal and X-linked intellectual developmental disorders and the exclusion of metabolic, neurodegenerative, and other genetic conditions capable of producing a similar combination of cognitive, behavioral, sensory, and systemic manifestations [3].

A major limitation of the present report is that it cannot establish a causal relationship between the patient's marfanoid features and *DLG4*-related synaptopathy. Although the clinical phenotype shows substantial overlap with previously described *DLG4*-associated manifestations, the identified *DLG4 *variant remains a variant of uncertain significance, and the absence of functional studies and familial segregation analyses precludes definitive assessment of its pathogenicity. Consequently, the proposed association between connective tissue manifestations and *DLG4 *dysfunction should be regarded as hypothesis-generating rather than confirmatory. Given that *DLG4*-related synaptopathy is a recently recognized and still incompletely characterized disorder, with limited data regarding its natural history, inheritance patterns, and genotype-phenotype correlations, additional well-characterized cases, functional validation, and segregation studies will be essential to determine whether marfanoid and connective tissue features represent part of the phenotypic spectrum of this condition.

## Conclusions

Our report contributes to the expanding phenotypic spectrum of *DLG4*-related disorders by describing a patient with extensive systemic involvement encompassing neurodevelopmental, musculoskeletal, psychiatric, sensory, and marfanoid manifestations, whose detailed clinical characterization underscores the importance of a comprehensive and integrated diagnostic approach in individuals presenting with overlapping connective tissue disorder-like, neurodevelopmental, and neuropsychiatric features. In such complex presentations, rare genetic disorders may remain underrecognized because patients are frequently evaluated separately by multiple specialties, emphasizing the need to interpret the clinical findings as part of a potentially unifying syndromic condition. Continued documentation of patients with phenotypes compatible with *DLG4*-related synaptopathy, together with molecular, functional, and familial investigations, may help refine current knowledge of its clinical heterogeneity, natural history, inheritance patterns, and genotype-phenotype correlations, thereby improving diagnostic recognition and clinical decision-making. Until disease-specific therapies become available, management should remain centered on individualized symptomatic treatment, supportive interventions, and coordinated multidisciplinary follow-up to preserve function and quality of life.
